# P02 Exploring medical students’ knowledge, attitudes, and motivation towards antimicrobial resistance efforts in Eastern Uganda

**DOI:** 10.1093/jacamr/dlae217.006

**Published:** 2025-01-23

**Authors:** Jonathan Babuya, Daniel Waruingi, Douglas Mungujakisa, Osmas Ahimbisibwe, Victoria Ruth Kako, Faith Aporu, Emmanuel Mugume, Julian Nyamupachitu, Kennedy Kiyimba

**Affiliations:** Faculty of Health Sciences, Busitema University, Mbale, Uganda; ReAct-Action on Antibiotic Resistance Africa, Nairobi, Kenya; Zihi Institute, Nairobi, Kenya; Faculty of Health Sciences, Busitema University, Mbale, Uganda; Faculty of Health Sciences, Busitema University, Mbale, Uganda; Faculty of Health Sciences, Busitema University, Mbale, Uganda; Faculty of Health Sciences, Busitema University, Mbale, Uganda; Faculty of Health Sciences, Busitema University, Mbale, Uganda; ReAct-Action on Antibiotic Resistance Africa, Nairobi, Kenya; Department of Pharmacology and Therapeutics, Faculty of Health Sciences, Busitema University, Mbale, Uganda

## Abstract

**Objectives:**

To assess knowledge, attitudes, and key motivational drivers influencing medical students’ participation in antimicrobial resistance (AMR)-related extracurricular initiatives at Busitema University, Uganda.

**Methods:**

A descriptive cross-sectional study was conducted among 193 undergraduate students. A semi-structured, pre-tested questionnaire was used to assess knowledge, attitudes, and motivations. Bloom cut-off method was used to evaluate knowledge, and χ^2^ and multivariate regression analyses were employed to identify engagement factors.

**Results:**

Overall, 71.5% of students had sufficient knowledge of AMR. Ninety percent supported integrating AMR into curricula, and 87.5% favoured a One Health Approach to training. Peer influence, university support and mentorship were top motivators for AMR club engagement. Male students and those in higher academic years were more engaged. Sufficient AMR knowledge was strongly associated with extracurricular involvement.

**Conclusions:**

Medical students demonstrated good knowledge and positive attitudes toward AMR, but deeper training is needed. Extracurricular activities play a pivotal role in motivating engagement. Embedding AMR and antimicrobial stewardship into curricula, along with peer mentorship, is critical for preparing future healthcare professionals to combat AMR.

